# Dietary supplementation with a designer metabolic modulator improves MASLD and associated anxiety in mice

**DOI:** 10.3389/fphar.2025.1661939

**Published:** 2025-10-13

**Authors:** Agnese Segala, Gaia Favero, Emanuela Bottani, Alice Vetturi, Emirena Garrafa, Edoardo Parrella, Chiara Ruocco, Maurizio Ragni, Rita Rezzani, Enzo Nisoli, Alessandra Valerio

**Affiliations:** ^1^ Department of Molecular and Translational Medicine, University of Brescia, Brescia, Italy; ^2^ Department of Clinical and Experimental Sciences, University of Brescia, Brescia, Italy; ^3^ Department of Laboratory Diagnostics, ASST Spedali Civili, Brescia, Italy; ^4^ Department of Engineering for Innovation Medicine, Section of Innovation Biomedicine, University of Verona, Verona, Italy; ^5^ Departmental Faculty of Medicine, Saint Camillus International University of Health Sciences, Rome, Italy; ^6^ Department of Medical Biotechnology and Translational Medicine, Center for Study and Research on Obesity, University of Milan, Milan, Italy

**Keywords:** metabolic dysfunction-associated steatotic liver disease, anxiety, mice, western diet, fibrosis, essential amino acids, tricarboxylic acid cycle intermediates, dietary supplement

## Abstract

**Background:**

Metabolic dysfunction-associated steatotic liver disease (MASLD), formerly known as non-alcoholic fatty liver disease (NAFLD), is a multifaceted condition characterized by excessive liver fat accumulation associated with obesity or other risk factors. Patients with obesity-related MASLD often suffer from comorbid psychiatric conditions, including anxiety. The therapeutic approach for MASLD relies on weight management through dietary and behavioral modifications. Nutritional interventions with essential amino acids (EAAs) have emerged as safe and promising tools in treating metabolic disorders and liver diseases. This study aimed to investigate the effects of dietary supplementation with α5, a designer EAA-based metabolic modulator enriched with tricarboxylic acid cycle intermediates, in a murine model of diet-induced MASLD with associated anxiety.

**Methods:**

Ten-week-old male C57BL/6J mice were fed for 17 weeks either a high-fat, high-sugar diet or a standard purified diet. The α5 compound (1.5 mg/g/day in drinking water) was administered to half of the mice fed each diet (n = 8/group). Mice body weight and energy intake were recorded. Liver and adipose tissue depot weights were calculated as ratios to body weight. Blood analytes were evaluated. Liver samples were analyzed for the enzymatic activity of mitochondrial chain respiratory complexes, gene expression (reverse transcription-qPCR), and histological features (hematoxylin-eosin and Masson’s trichrome staining). Liver disease severity was graded using the NAFLD Activity Score. The open field behavioral test was conducted to assess anxiety.

**Results:**

Mice fed the high-fat, high-sugar diet developed obesity, a MASLD phenotype, and anxiety-like behaviors. Dietary supplementation with α5 ameliorated liver pathology, including reduced hepatocellular ballooning, fat lipid droplet diameter, and the expression of genes related to fibrosis, without affecting body weight. Moreover, α5 supplementation significantly reduced the anxiety-like behavior observed in untreated MASLD mice.

**Discussion:**

These results suggest that α5 represents a novel intervention to prevent or mitigate the progression of MASLD and its associated mental health complications.

## 1 Introduction

Metabolic dysfunction-associated steatotic liver disease (MASLD), formerly known as non-alcoholic fatty liver disease (NAFLD), is a progressive condition characterized by triglyceride (TG) liver accumulation in the absence of excessive alcohol intake (Tack et al., 2024). The updated diagnostic criteria emphasize both hepatic fat content exceeding 5%–10% of liver weight and the presence of at least one risk factor among overweight or obesity, hyperglycemia or type 2 diabetes, elevated TGs, reduced HDL-cholesterol, or increased blood pressure (Tack et al., 2024). MASLD affects approximately 30% of the global population, a prevalence that continues to rise alongside the spread of unhealthy lifestyles, including Western-type diets and physical inactivity (Younossi et al., 2025). The disease spectrum ranges from isolated steatosis (termed metabolic dysfunction-associated steatotic liver, MASL) to more advanced stages such as steatohepatitis (MASH), which features hepatic inflammation and hepatocyte ballooning. If untreated, MASH can progress to fibrosis, cirrhosis, and ultimately hepatocellular carcinoma (Huang et al., 2025). A major clinical challenge is the typically silent early phase of MASLD, during which symptoms are either absent or nonspecific, leading to delayed diagnosis (Eskridge et al., 2023).

Despite growing research efforts, the pathophysiological mechanisms of MASLD remain incompletely understood. Known contributors include genetic predisposition and excessive intake of dietary fat and sugar, which promote adipocyte dysfunction, leading to the release of free fatty acids and ectopic accumulation of lipids in the liver (Huang et al., 2025). In parallel, increased hepatic *de novo* lipogenesis and mitochondrial dysfunction contribute to oxidative stress and impaired ATP production, ultimately leading to hepatocellular injury, chronic inflammation, and fibrosis (Huang et al., 2025; Radosavljevic et al., 2024).

Emerging evidence also links MASLD to neuropsychiatric comorbidities. Patients with MASLD/NAFLD exhibit higher rates of anxiety and depression (Hadjihambi et al., 2023; Labenz et al., 2020; Shea et al., 2024; Wang et al., 2024), even in the early, pre-diagnosis stages of the disease (Eskridge et al., 2023). These associations appear to be multifactorial and bidirectional, involving insulin resistance, low-grade chronic inflammation, and alterations in the gut microbiome (Shea et al., 2024). Possibly perpetuating a detrimental cycle between metabolic dysfunction and mental health (Shea et al., 2024).

Currently, resmetirom (a thyroid hormone β-receptor agonist) is the only approved pharmacological treatment for MASH with advanced fibrosis. Still, other therapeutic agents have demonstrated promising results in randomized controlled trials (Huang et al., 2025; Lin et al., 2024). Due to the complex nature of MASLD/MASH, combination therapies are also under investigation (Dufour et al., 2020). For now, weight-loss programs, including the adoption of healthy dietary patterns and lifestyle modification, represent the cornerstone of MASLD treatment (Segala et al., 2024). Unfortunately, adherence to such interventions is generally low among patients with MASLD (Frith et al., 2010), and the presence of comorbid anxiety may further reduce compliance.

Our research group has long-standing experience in supplementing diets with essential amino acids (EAAs). We extensively investigated an EAA balanced formula, termed branched-chain amino acid-enriched mixture (BCAAem), which enhances mitochondrial metabolism and the endogenous antioxidant response, exerting beneficial effects in various organs and pathophysiological conditions (see Ruocco et al., 2021, for a review). Of note, we previously demonstrated that the BCAAem prevents liver steatosis in a rodent model of alcoholic liver disease (Tedesco et al., 2018). Building on our previous work, we recently designed novel EAA-based metabolic modulators incorporating tricarboxylic acid (TCA) cycle intermediates, which showed superior efficacy in promoting mitochondrial function compared to the original BCAAem formulation (Brunetti et al., 2020; Ruocco et al., 2021). One such compound, referred to as α5, consists of eleven EAAs, including balanced stoichiometric ratios of the BCAAs (leucine:isoleucine:valine ratio, 3:1:1), enriched with three TCA cycle intermediates (citric, malic, and succinic acids) and cofactors (Ruocco et al., 2021; Tedesco et al., 2020). The α5 compound has been shown to support neuronal energy metabolism *in vitro* and *in vivo* (Bifari et al., 2020; Dolci et al., 2022), suggesting its safety and therapeutic potential not only in metabolic disease, but also in neuropsychiatric disorders.

In this study, we fed adult mice a high-fat, high-sugar diet (HFHSD) that was previously shown to induce a MASLD/MASH phenotype closely resembling the human disease (Verbeek et al., 2015). Fare clic o toccare qui per immettere il testo. Our findings demonstrate that dietary supplementation with the designer metabolic modulator α5 improves liver pathology and alleviates anxiety-like behavior in mice with a Western-type diet-induced MASLD.

## 2 Materials and methods

### 2.1 Animals, diets, and treatment

**FIGURE 1:**
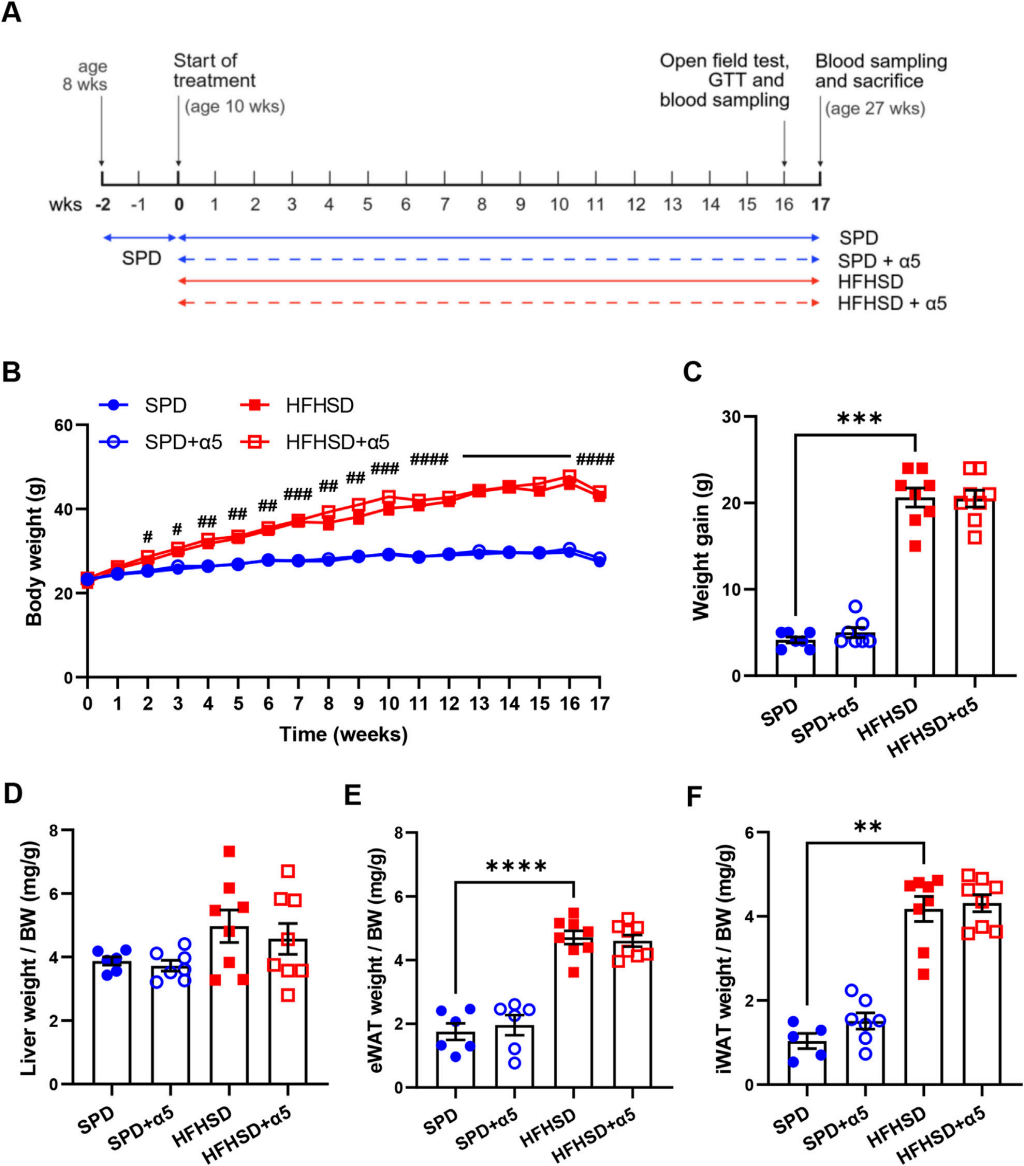
Effects of the nutritional interventions on body and organ weight. **(A)** Schematic representation of the experimental design timeline. Ten-week-old C57BL/6J mice were fed *ad libitum* with standard purified diet (SPD) or high-fat, high-sugar diet HFHSD with or without α5 supplementation for 17 weeks. Image was created with BioRender.com. **(B)** Weekly measurement of body weight; **(C)** Body weight gain, measured as the difference between the final and initial (at the start of the treatment) body weight. **(D)** Liver weight normalized to body weight (BW); **(E,F)** weight of visceral (eWAT, epididymal white adipose tissue) and subcutaneous (iWAT, inguinal WAT) fat pads normalized to BW. Data represent mean ± SEM (n = 5-8 mice/group; missing points are due to technical issues). Statistical analysis was performed by the two-way ANOVA, followed by Tukey’s multiple comparisons test **(B)**; the ordinary one-way ANOVA, followed by Šídák’s multiple comparisons test **(D,E)**, or the Kruskal-Wallis test, followed by Dunn’s multiple comparison test **(C-F)**. **p < 0.01, ***p < 0.001 and ****p < 0.0001; #p < 0.05, ##p < 0.01, ###p < 0.001, ####p < 0.0001 in HFHSD vs SPD.

All experiments were performed in accordance with the European Directive 2010/63/EU and current Italian law (D. Lgs. n. 26/2014). The protocol was approved by the General Direction of Animal Health and Veterinary Drugs of the Italian Ministry of Health with the authorization n. 498/2018-PR. Eight-week-old male C57BL/6J mice (n = 32) (Charles River, Calco, Italy) were housed under controlled temperature and humidity conditions, in a 12-h light-dark cycle, with *ad libitum* access to food and water. After 2 weeks of acclimatization, mice were randomized into four groups (n = 8/group, 4/cage) and fed for 17 weeks with either Standard Purified Diet (SPD; AIN-93M formula, TD.94048, Envigo, Italy) or HFHSD (TD.08811, Envigo, Italy) (Supplementary Table S1). The length of the dietary intervention was based on previous reports using the same HFHSD (Gariani et al., 2016; Verbeek et al., 2015). Half of the mice fed each of the two diets were supplemented with the α5 (Professional Dietetics S. p.A, Milan, Italy) for the entire experiment duration (Figure 1A). Body weight and food intake were recorded weekly, and drinking volume was measured three times a week. The composition of the α5 supplement is detailed in Supplementary Table S2. It was administered at a dose of 1.5 mg/g body weight/day in drinking water, as previously described (Tedesco et al., 2020), and the solution was replaced three times a week. The amount of α5 to be dissolved in water was calculated for each experimental group based on the average body weight from the most recent measurement and the average daily water consumption over the previous 2 weeks. This calculation was adjusted regularly according to these parameters. Blood was collected from the tail vein at week 16 or via submandibular venipuncture immediately before sacrifice. All blood drawings were performed after a 7-h fast. At the end of the study, mice were euthanized by cervical dislocation. Tissue, serum, and plasma samples were collected and snap-frozen in liquid nitrogen, then stored at −80 °C. One mouse in the SPD group died soon after randomization. One mouse in the SPD+α5 group showed a suspected liver neoplasm at sacrifice, and its data were excluded from analyses.

### 2.2 Behavioral tests

The open field test was used to assess anxiety as described (Keleher et al., 2018) with minor modifications. The arena consisted of a plastic box [40 (length) × 40 (width) × 40 (height) cm] with a grid on the floor to identify a 25 × 25 cm area defined as the central zone. The test was conducted in a quiet, dimly lit room, during the light phase of the light-dark cycle between 9 a.m. and 4 p.m. Briefly, mice were individually placed in the center of the arena and allowed to explore freely for 5 min. Mice’s behavior was recorded and analyzed by a video tracking system (ANY-Maze, Stoelting Co., IL, United States). The following measurements were collected: total distance traveled, average speed, time spent moving, time spent in the peripheral zone, number of entries into the central zones, and number of fecal boli produced. Preference to stay in the peripheral zones, reduced visits to the central zone, and increased fecal boli production were interpreted as indicators of anxiety.

### 2.3 Circulating analytes and glucose homeostasis evaluation

Blood samples for serological analyses and assessment of glucose homeostasis were collected from the tail vein after a 7-h fast at week 16 of treatment. Serum levels of insulin, C-reactive protein (CRP), and tumor necrosis factor-alpha (TNF-α) were measured by ELISA immunoassay. We used Mouse Insulin ELISA Kit (ab277390, Abcam, Cambridge, United Kingdom), Mouse C Reactive Protein ELISA Kit (PTX1) (ab157712, Abcam, Cambridge, United Kingdom), Mouse TNF-alpha Quantikine HS ELISA (MHSTA50, R&D Systems, Bio-techne, Minneapolis, MN, United States), respectively, according to the manufacturers’ instructions. For the glucose tolerance test (GTT), mice received an intraperitoneal (i.p.) injection of glucose (0.75 mg/g body weight; Sigma-Aldrich, Milan, Italy) (n = 4 mice/group). Blood glucose concentrations were measured from the tail vein at baseline (0 min) and at 15, 30, 60, and 120 min post-injection using the OneTouch Verio Reflect glucometer (LifeScan, Sesto San Giovanni, Italy) (Ruocco et al., 2020). The area under the curve (AUC) was calculated using the trapezoidal rule (Ruocco et al., 2020). Blood samples for biochemical analysis were obtained via submandibular venipuncture before sacrifice, collected in EDTA-containing tubes, and then centrifuged to separate the plasma. The following analytes were measured: total cholesterol, TGs, alanine aminotransferase (ALT), and aspartate aminotransferase (AST). The homeostasis model assessment of insulin resistance (HOMA-IR) index was calculated by multiplying fasting insulin (ng/mL) by fasting glucose (mg/dL) and dividing the result by 405 (Verbeek et al., 2015).

### 2.4 Mitochondrial respiratory complex activities

Homogenates of frozen mouse liver samples were prepared as described by Spinazzi and colleagues (Spinazzi et al., 2012), with minor modifications (Brunetti et al., 2020). Enzymatic activities of individual MRC complexes were measured spectrophotometrically. Assays of complex I (CI, NADH:ubiquinone reductase), II (CII, succinate dehydrogenase), III (CIII, decylubiquinol cytochrome c oxidoreductase), and IV (cytochrome *c* oxidase), as well as the citrate synthase assay, were performed as described in detail (Spinazzi et al., 2012). The complex V (CV, F_1_-ATPase) assay was performed according to Frazier and Thornburn (Frazier and Thorburn, 2012). All enzymatic activities were normalized to the citrate synthase activity.

### 2.5 Reverse transcription and quantitative PCR

For the analysis of mRNA expression, total RNA was isolated using the RNeasy Plus Mini Kit (Qiagen, Hilden, Germany). RNA concentration and purity were assessed with the NanoDrop™ OneC Microvolume UV-Vis Spectrophotometer (Thermo Scientific, Milan, Italy). Total RNA (2 µg) was reverse transcribed using iScript cDNA Synthesis Kit (Bio-Rad Laboratories, Segrate, Italy). cDNA was diluted 1:5 in DNAse-free water, and 2 µL were amplified by real-time quantitative PCR with iTaq Universal SYBR Green SuperMix (Bio-Rad Laboratories) on a ViiA7 Real-Time PCR system (Applied Biosystems). Each sample reaction was conducted in triplicate. Primer sequences (Supplementary Table S3) were designed using Primer3 software (version 0.4.0). Relative gene expression was calculated by a comparative method (2^−ΔΔCT^). Hypoxanthine-guanine phosphoribosyltransferase (HPRT) was used as a housekeeping gene after evaluating its stable expression. To measure mtDNA content, total DNA was extracted using the QIAamp DNA Mini Kit (Qiagen). Then, qPCR was performed using primers specific for the mitochondrially encoded gene Cytochrome c oxidase subunit I (*Co1*) and the nuclear gene Ribonuclease P (*Rnase P*) (Supplementary Table S3). The mtDNA copy number (mtDNAcn) was calculated as previously described (Segala et al., 2024).

### 2.6 Liver morphometrical analyses and triglyceride content assay

Liver samples were fixed in a 4% paraformaldehyde solution for 48 h and embedded in paraffin wax. Serial sections (5 µm thick) were cut with a semiautomatic microtome. Alternate sections were deparaffinized, rehydrated, and stained with hematoxylin-eosin (H&E) to assess overall liver parenchymal morphology, as well as liver and hepatocyte abnormalities (including ballooning, steatosis, and inflammatory infiltration), and the diameter of lipid droplets (Raffaele et al., 2019). Masson’s trichrome staining was performed to evaluate liver fibrosis, with fibrotic connective tissue stained blue, and the hepatocyte cytoplasm red (Raffaele et al., 2019). Sections were observed with an optical light microscope (Olympus BX50, Hamburg, Germany) at a final magnification of ×200. Image analysis was performed using the Image Pro Premier 9.1 software program (Media Cybernetics, Rockville, MD, United States). Lipid droplet diameter was measured by evaluating a minimum of 10 droplets per randomly chosen field on five non-consecutive sections per mouse. In addition, a frequency analysis of lipid droplets was performed based on arbitrary diameter clustering into the following five classes: <3.5 µm, between 3.5 and 7 μm, between 7 and 15 μm, between 15 and 25 μm, and >25 µm. The presence and percentage of fibrosis were measured in five randomly chosen fields per mouse. All analyses were evaluated in a single-blind manner by an experienced examiner. Liver disease severity was histologically assessed according to the NAFLD activity score (NAS) system as follows: degree of steatosis (grade 0 ≤ 5%; grade 1 = 5–33%; grade 2 = 34%–66%; grade 3 ≥ 66%), inflammation (0: no foci, 1 < 2 foci per 200x field, 2: 2 to 4 foci per 200x field, and 3: >4 foci per 200x field) and ballooning (0: none; 1: rare or few; 2: many). The NAS is the sum of these indices (Verbeek et al., 2015). The hepatic TG content was measured with the Triglyceride Colorimetric Assay Kit (10010303, Cayman Chemical, Ann Arbor, MI, United States) using a standard curve according to the manufacturer’s instructions.

### 2.7 Statistical analysis

The normality of the data was assessed with the Shapiro-Wilk test. Data without a Gaussian distribution were analyzed using the non-parametric Kruskal-Wallis test, followed by Dunn’s multiple comparison test; data with a Gaussian distribution were analyzed with an Ordinary One-way ANOVA test, followed by Šídák’s multiple comparisons test. The following comparisons were performed: SPD vs. SPD + α5, SPD vs. HFHSD, and HFHSD vs. HFHSD + α5. Behavioral test parameters were analyzed using the Mann-Whitney test if they were non-normal, or the unpaired t-test if they were normally distributed. Data were expressed as mean ± SEM. A p-value <0.05 was considered statistically significant. Outliers were identified by the ROUT or Grubbs’ methods. Statistical analyses and graphs were performed using Prism 9.0.0 (GraphPad, La Jolla, California, United States).

## 3 Results

### 3.1 Dietary α5 supplementation does not affect mice body weight and fat depots

We first characterized weight-related changes in male C57BL/6J mice during 17 weeks of nutritional interventions. Starting at week 2, C57BL/6J mice fed HFHSD showed a progressive, significant increase in body weight compared to SPD-fed mice (Figure 1B). Consistently, we observed a significantly higher weight gain (Figure 1C), supported by a higher daily average calorie intake, in the HFHSD-compared to the SPD-fed group (Supplementary Figure S1B). The weight increase in HSHSD-fed mice was associated with augmented depots of visceral and subcutaneous white adipose tissues (WAT), as measured by body weight-normalized epididymal and inguinal WAT weight (Figures 1E,F). These changes occurred with a slight, non-significant increase in liver weight relative to body weight in HFHSD-fed mice compared to the SPD group (Figure 1D). Administration of the α5 compound reduced food intake in SPD- but not in HFHSD-fed mice (Supplementary Figure S1A) with no changes in energy intake (Supplementary Figure S1B), and increased water intake both in SPD- and HFHSD-fed mice (Supplementary Figure S1C). Supplementation with α5 did not significantly affect body weight, body weight gain, or body weight-normalized organ weight in SPD- nor HFHSD-fed mice (Figures 1B–F).

### 3.2 Dietary α5 supplementation does not affect biomarkers of glycemic or lipid homeostasis

**FIGURE 2:**
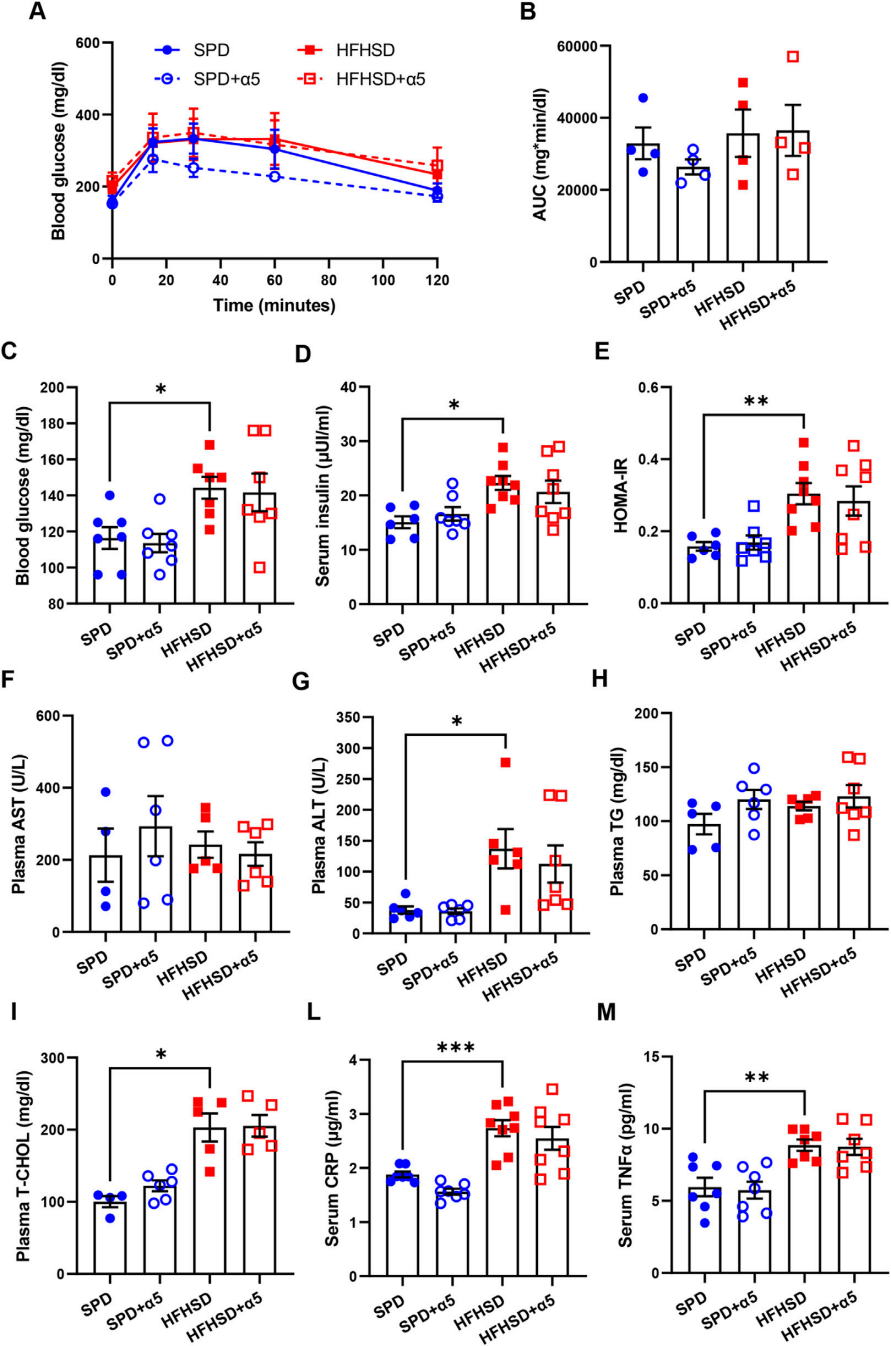
Assessment of blood biochemical analytes and inflammatory biomarkers. **(A,B)** Blood glucose after a glucose tolerance test; the panel on the right **(B)** shows the area under the curve (AUC). **(C,D)** Blood glucose and serum insulin levels; **(E)** homeostasis model assessment of insulin resistance (HOMA-IR) index; **(F–I)** plasma aspartate aminotransferase (AST), alanine transaminase (ALT), triglycerides (TG), total cholesterol (T-CHOL) levels; **(L,M)** serum C-reactive protein (CRP) and tumor necrosis factor-α (TNFα) levels. All analytes were measured after a 7-h fast. Data represent mean ± SEM (n = 4-8 mice/group; missing points are due to technical issues). Outliers were excluded by the ROUT method. Statistical analyses were performed using one-way ANOVA, followed by Šídák’s multiple comparisons test **(B–H,L,M)** or the Kruskal-Wallis test, followed by Dunn’s multiple comparison test **(G-I)** and. *p < 0.05, **p < 0.01 and ***p < 0.001.

As expected (Verbeek et al., 2015), feeding mice with HFHSD induced insulin resistance, characterized by significantly increased glycemia and insulinemia, as well as a higher HOMA-IR index, compared to the SPD (Figures 2C–E). This finding was not accompanied by a worse response of HFHSD-fed mice to the GTT, measured at week 16 after a 7-h fast (Figures 2A,B). The 17-week HFHSD also altered lipid homeostasis, with significantly increased total cholesterol levels (Figure 2I), but no difference in TG levels (Figure 2H), as previously shown (Verbeek et al., 2015). Compared to mice fed SPD, mice fed HFHSD for 17 weeks exhibited hepatic damage, as indicated by strongly increased circulating ALT levels (Figure 2G) and an inflammatory status, characterized by elevated serum CRP and TNFα levels (Figures 2L,M).

Dietary supplementation with α5 elicited a non-significant improvement in glucose tolerance in SPD-fed mice (Figures 2A,B). The α5 compound did not alter the levels of the dosed biochemical analytes in either SPD-fed or HFHSD-fed mice (Figures 2C–I). A visible but non-significant trend towards reduced ALT and CRP levels was found in mice fed HFHSD when supplemented with α5 (Figures 2G-L).

### 3.3 Dietary α5 supplementation attenuates HFHSD-induced fatty liver disease

**FIGURE 3:**
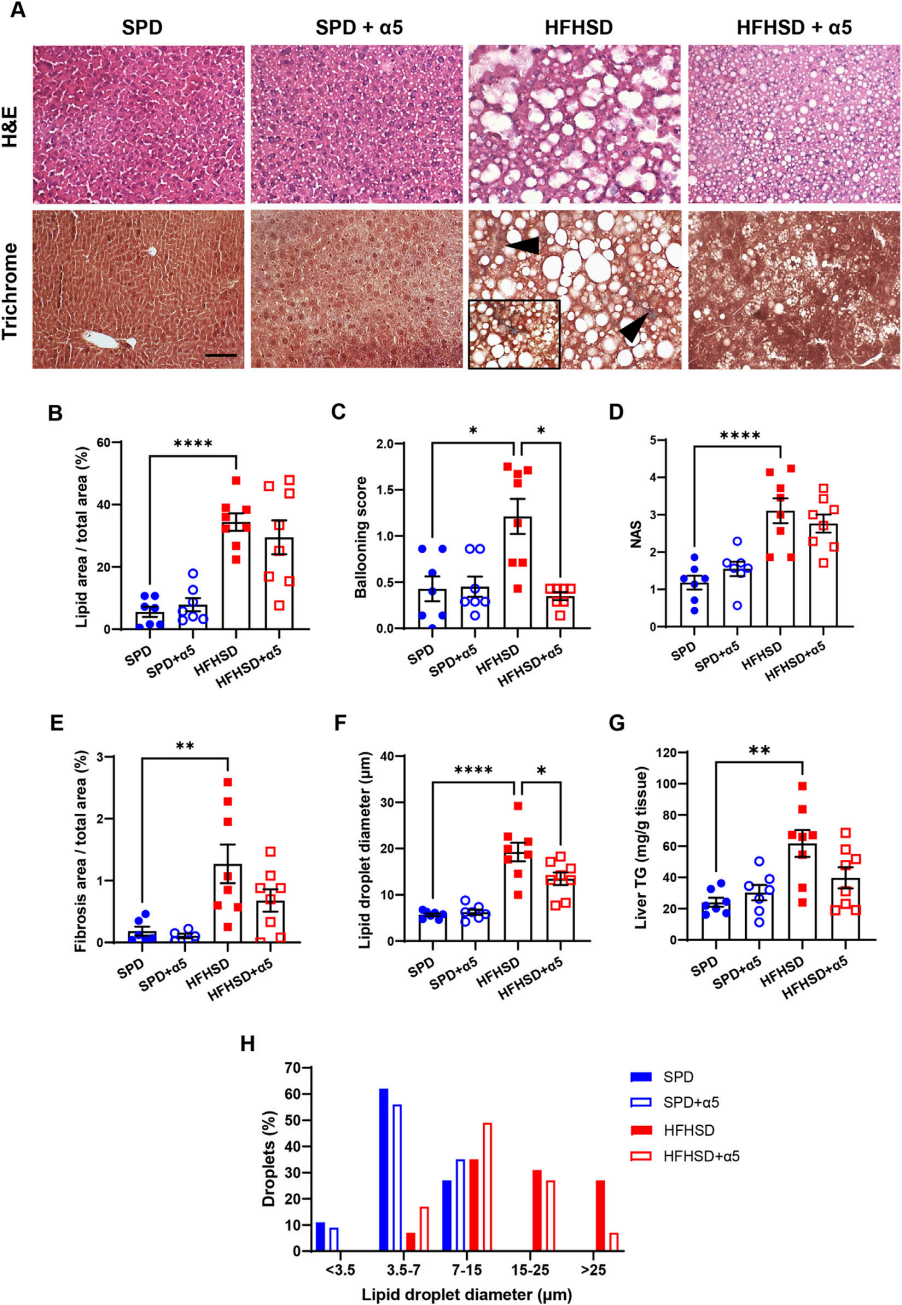
Liver histological analyses. **(A)** Representative photomicrographs of hematoxylin and eosin (H&E) or Masson’s trichrome-stained specimens of mouse liver. Arrowheads and 40× frame denote areas of blue-colored collagen, indicating fibrosis. Bar, 50 µm. **(B–F)** Morphometric analysis parameters: percent steatosis measured as lipid area/total area; ballooning score; NAFLD activity score (NAS); percent fibrosis measured as collagen area/total area; lipid droplet diameter. **(G)** Frequency distribution of lipid droplet diameter classes. Data represent mean ± SEM (n = 5-8 mice/group). Outliers were excluded by the ROUT method. The Statistical analysis was performed using one-way ANOVA, followed by Šídák’s multiple comparisons test **(B,D–F)** or the Kruskal-Wallis test, followed by Dunn’s multiple comparison test **(C)**.*p < 0.05, **p < 0.01, and ****p < 0.0001.

Liver histology was performed to assess parenchymal architecture and associated pathological changes. Representative images are presented in Figure 3A. Mice fed SPD displayed occasional hepatocyte ballooning but no signs of steatosis, inflammatory infiltration, or fibrosis. In contrast, after 17 weeks on HFHSD, mice developed marked hepatic steatosis and pronounced hepatocyte ballooning (swollen hepatocytes containing large lipid droplets and peripherally displaced nuclei) along with sporadic lobular inflammation and mild fibrosis. While α5 supplementation did not alter hepatic histology in SPD-fed mice, it improved liver morphology in HFHSD-fed mice, which exhibited fewer and smaller lipid droplets, as well as decreased steatosis and ballooning. Morphometric analysis confirmed significant increases in lipid area, hepatocyte ballooning, fibrosis area, and histological score of fatty liver disease severity (NAS) in HFHSD-fed mice compared to the SPD-fed ones (Figures 3B–E). The diameter of hepatic lipid droplets was also significantly greater in the HFHSD group (Figure 3F), with 26% of droplets displaying a diameter >25 μm (Figure 3H). These results were accompanied by a significant increase in the hepatic amount of TG in HFHSD-fed mice (Figure 3G). The α5 supplementation showed only a trend towards improvement of some parameters (Figures 3B,D,E), including a non-significant but consistent amelioration of liver TG content (p = 0.0581 vs HFHSD) (Figure 3G). Notably, α5 supplementation was significantly effective in reducing both hepatocyte ballooning (Figure 3C) and lipid droplet diameter (Figure 3F). The treatment increased the percentage of droplets with a diameter between 3.5 and 15 μm and reduced that of droplets with a diameter ≥25 μm (Figure 3H). Collectively, these findings indicate that α5 acts as a metabolic modulator capable of ameliorating diet-induced MASLD in mice.

### 3.4 Treatment with α5 supports hepatic mitochondrial activity in HFHSD-fed mice

**FIGURE 4:**
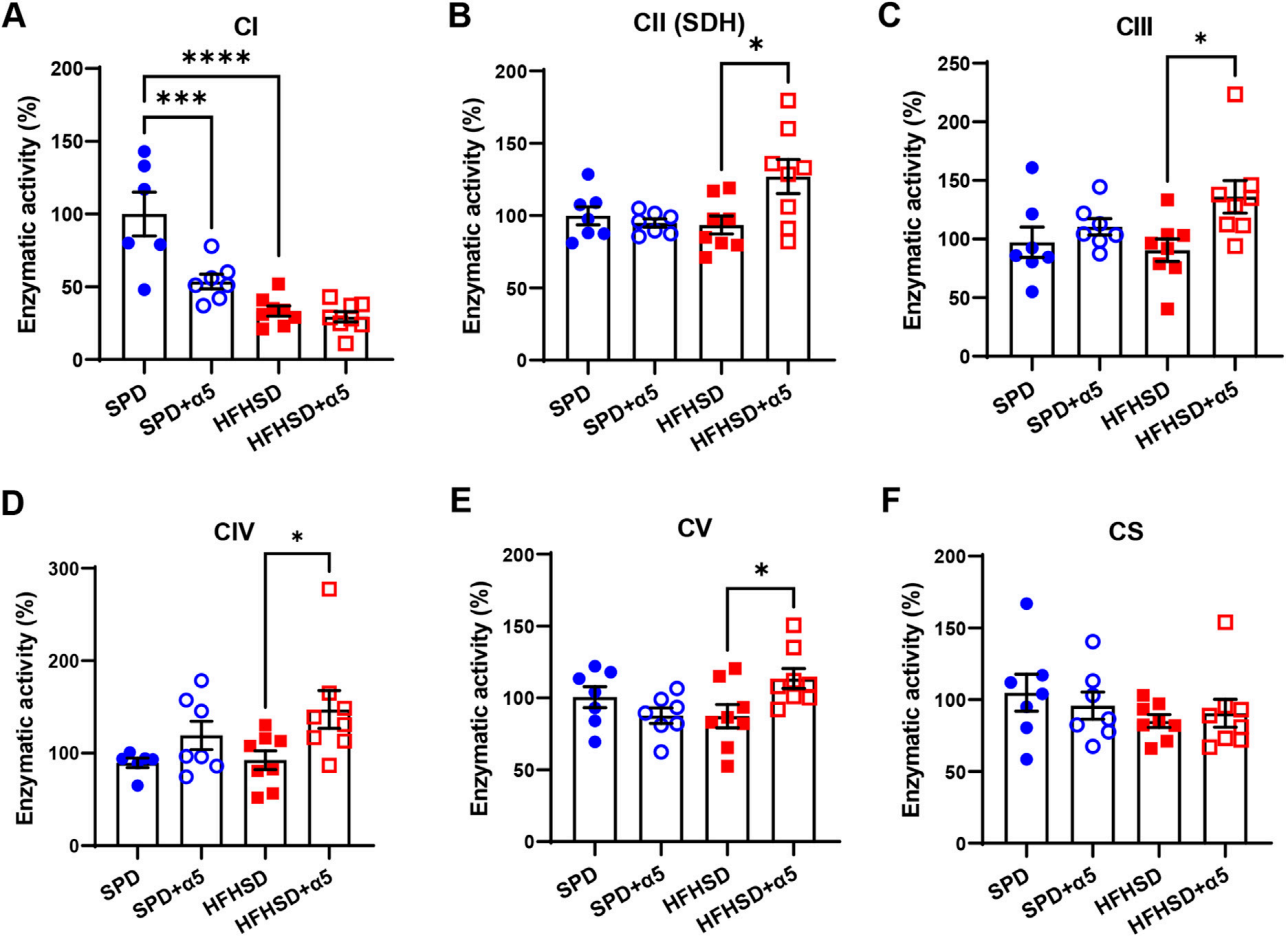
Assessment of liver mitochondrial function. **(A–F)** Enzymatic activities of the mitochondrial respiratory chain complex I (CI), II (CII), III (CIII), IV (CIV), and citrate synthase (CS). Data represent mean ± SEM (n = 6-8 mice/group). One outlier was excluded by the ROUT method. The statistical analysis was performed using one-way ANOVA, followed by Šídák’s multiple comparisons test **(A–C,E)** or the Kruskal-Wallis test, followed by Dunn’s multiple comparison test **(D-F)**. *p < 0.05, ***p < 0.001 and ****p < 0.0001.

Analysis of the enzyme activity of mitochondrial respiratory chain complexes in liver showed a strongly reduced CI activity in HFHSD-fed compared to SPD-fed mice (Figure 4A), as previously described (Verbeek et al., 2015). The activity of the other liver respiratory chain complexes remained unchanged after a 17-week HFHSD under our conditions. Unexpectedly, we observed reduced hepatic CI activity in SPD-α5-treated mice when compared to SPD mice, without changes in the HFHSD-α5 vs the HFHSD group (Figure 4A). Treatment with α5 did not affect other MRC complexes in SPD-fed mice (Figures 4B–E). Of interest, the α5 supplement significantly increased the CII, CIII, CIV, and CV activities in livers from HFHSD-fed mice (Figures 4B-E). The enzymatic activity of citrate synthase was not modified by any treatment (Figure 4F), suggesting unchanged mitochondrial mass. Accordingly, neither HFHSD nor α5 supplementation altered the mRNA levels of peroxisome proliferator-activated receptor γ coactivator 1α (*PGC-1α*) or mtDNA content in the liver (Supplementary Figure S2).

### 3.5 Dietary α5 supplementation reverses the HFHSD-mediated increase in fibrosis markers

**FIGURE 5:**
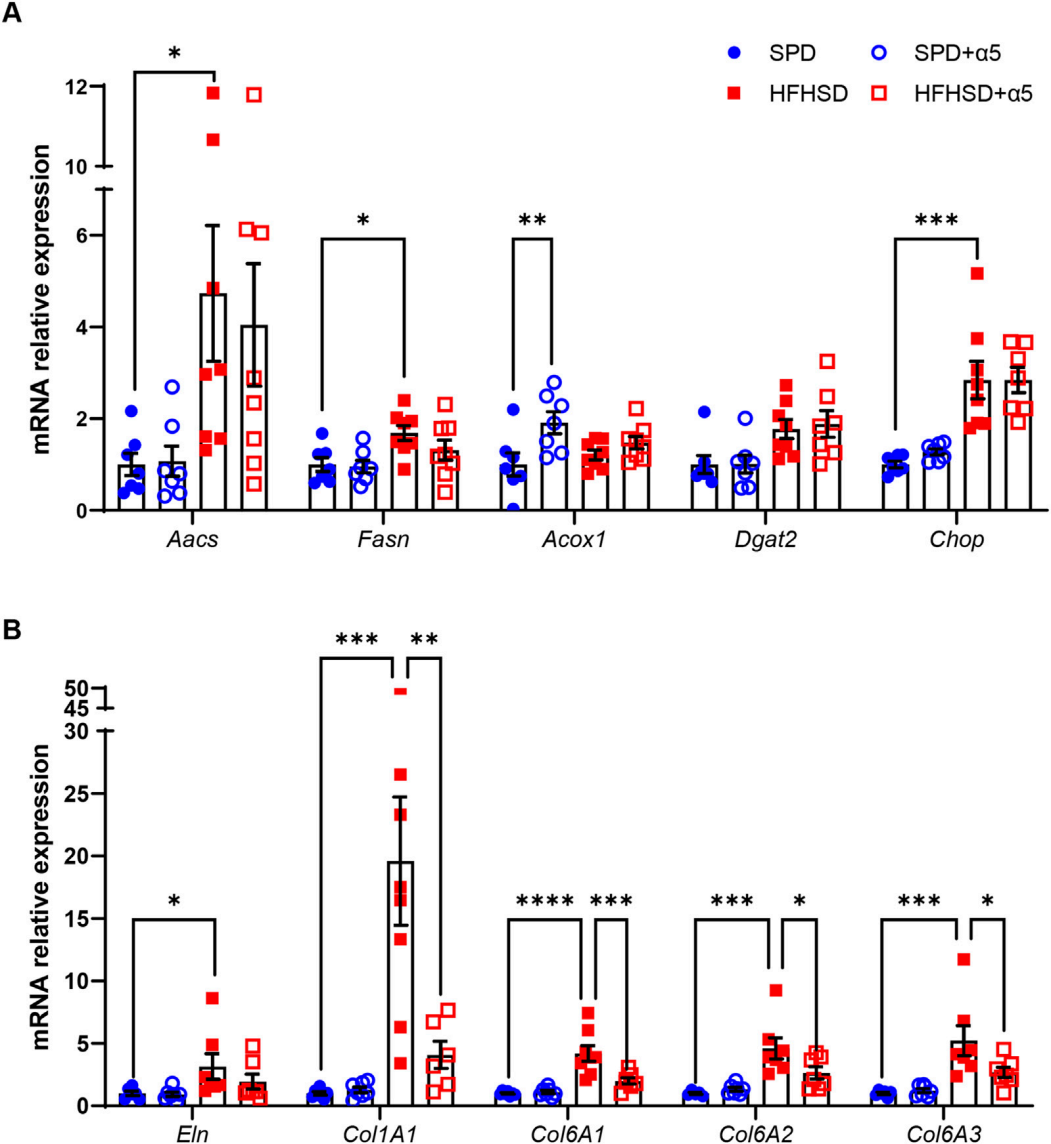
Relative mRNA expression in mouse liver. Reverse transcription-qPCR data of **(A)** Acetoacetyl-CoA Synthetase (*Aacs*), Fatty acid synthase (*Fasn*), Acyl-CoA oxidase 1 (*Acox1*), Diacylglycerol O-acyltransferase 2 (*Dgat2),* and C/EBP homologous protein (*Chop*); **(B)** Elastin (*Eln*), Collagen type I alpha 1 (*Col1A1*), Collagen type VI alpha 1 (*Col6A1*), Collagen type VI alpha 2 (*Col6A2*), and Collagen type VI alpha 3 (*Col6A3*). Data were normalized to *Hprt* expression. Values are reported as relative expression compared to the SPD group, taken as 1. Data represent mean ± SEM (n = 5-8 mice/group). Outliers were excluded by the Grubbs’ method. The statistical analysis was performed by the Kruskal-Wallis test, followed by Dunn’s multiple comparison test *(Aacs, Dgat2, Chop, Eln*)) or one-way ANOVA, followed by Šídák’s multiple comparisons test (all other mRNAs). *p < 0.05, **p < 0.01, ***p < 0.001 and ****p < 0.0001.

We investigated the changes in gene expression that may be altered in diet-induced MASLD. The mRNA levels of diacylglycerol O-acyltransferase 2 (*Dgat2*), a key player in liver TG synthesis, appeared slightly but non-significantly augmented by HFHSD. Still, those of two key enzymes involved in *de novo* lipogenesis, the acetoacetyl-CoA synthetase (*Aacs*) and the fatty acid synthase (*Fasn*), were significantly increased in the liver from HFHSD-fed compared to the SPD-fed group (Figure 5A). In addition, increased mRNA levels of the endoplasmic reticulum (ER) stress marker C/EBP homologous protein (*Chop*) were observed in the HFHSD mice compared to the SPD group (Figure 5A). Supplementation with α5 did not modify these lipogenesis or ER stress markers in HFHSD-fed mice (Figure 5A). While the expression of the acyl-CoA oxidase 1 (*Acox1*), which promotes peroxisomal β-oxidation and catabolism of very long-chain fatty acids (VLCFAs), was not affected by HFHSD, α5 administration *per se* induced a significant increase in *Acox1* mRNA levels in mice fed with SPD (Figure 5A). Livers from HFHSD-fed mice displayed augmented expression of genes involved in fibrogenic pathways. Elastin (*Eln*) mRNA levels were increased, with a trend toward reduction in mice treated with α5. Further, collagen type I alpha 1 (*Col1A1*) mRNA was induced 15-fold, and *Col6A1*, *Col6A2*, and *Col6A3* mRNAs were significantly increased in the HFHSD group (Figure 5B). Notably, dietary supplementation with α5 almost completely reversed the increase of all the collagen types investigated (Figure 5B).

### 3.6 The anxious behavior of MASLD mice is prevented by α5 supplementation

**FIGURE 6:**
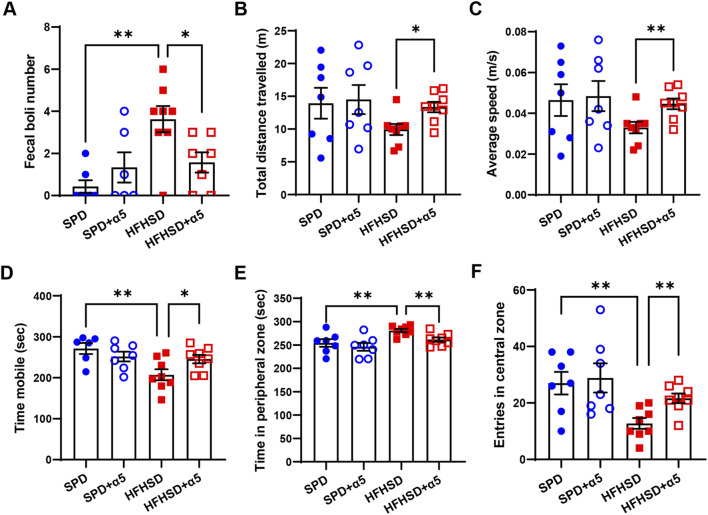
Characterization of the anxiety behavior in HFHSD-fed mice with and without α5 supplementation. **(A)** Fecal boli number count in the arena at the end of the test; **(B–F)** Open field test parameters. Data represent mean ± SEM (n = 6-8 mice/group). One outlier was excluded by the ROUT method. The unpaired t-test (B–F) and the Mann-Whitney test **(A)** were used for statistical analysis. *p < 0.05 and **p < 0.01.

We assessed the behavioral performance of mice at the 16th experimental week. The open field test was adopted to evaluate anxiety status as thigmotaxis (i.e., the tendency to remain in a protected area close to the arena walls) and defecation level (Bourin et al., 2007). Representative track plots of mice movements in the arena are shown in Supplementary Figure S3. We observed an increase in the number of fecal boli (Figure 6A) and a corresponding increase in time spent in the peripheral zone of the arena, accompanied by a significant reduction in time spent mobile and the number of entries in the central zone in HFHSD-fed mice compared to SPD-fed mice (Figures 6D–F). Although not statistically significant, a downward trend in total distance traveled and average speed of movement was also observed (Figures 6B,C). Together, these data demonstrate that HFHSD-fed mice experience anxiety symptoms. When compared to unsupplemented HFHSD mice, α5-treated HFHSD mice exhibited significantly increased total distance travelled, average speed, mobility time, and number of entries in the central zone, as well as reduced number of fecal boli and time spent in the peripheral zone. Thus, our results support the efficacy of α5 in rescuing anxious symptoms in a fatty liver mouse model obtained by HFHSD feeding.

## 4 Discussion

Despite various promising preclinical and clinical studies, MASLD remains an unmet medical need. Weight loss achieved through diet and lifestyle modifications is the primary approach for patients in the early stage of the disease, i.e., those presenting signs of liver steatosis (MASL) without evidence of fibrosis. However, early treatment is of paramount importance in reducing liver fat deposition and preventing disease progression to advanced stages with severe fibrosis (MASH). Hence, novel therapeutic strategies are needed. In the present work, we investigated the efficacy of a nutritional intervention based on the α5 metabolic modulator in a mouse model of diet-induced MASLD.

After 17 weeks on the HFHSD, mice developed obesity, with increased weight of subcutaneous and visceral fat depots, and insulin resistance (as indicated by an increased HOMA-IR index). Their unchanged glucose tolerance could be related to the smaller group size in the GTT experiment. Mice under HFHSD also exhibited hypercholesterolemia, without differences in triglyceridemia. Though we used a different reference diet (i.e., SPD instead of chow diet) and introduced minor modifications in the experimental plan, these results are consistent with those reported with the same Western-type diet by Verbeek and collaborators (Verbeek et al., 2015). The systemic metabolic effects induced by HFHSD were accompanied by increased plasma ALT (an indicator of liver damage), augmented hepatic TG content, liver histological abnormalities (steatosis, hepatocyte ballooning, and fibrosis), and a higher histological score for the severity of fatty liver disease (NAS), which recapitulated the advanced stages of MASLD. Reduced CI enzymatic activity completed the picture. Notably, mice on the HFHSD also exhibited behavioral alterations, characterized by decreased exploratory activity in the open field test. Clear signs of an anxiety state (Bourin et al., 2007) included augmented defecation episodes during the test, spending more time moving in the periphery of the arena, and entering the center area fewer times. Thus, this Western diet model is well-suited to investigating the effects of novel therapeutic approaches to liver pathology and coexisting anxiety symptoms.

Our data demonstrate that dietary supplementation with α5 is efficacious against HFHSD-related MASLD, with no effect on energy intake or body weight. The intervention did not alter glycemic or lipidic homeostasis, nor modify any biochemical analyte in either SPD or HFHSD conditions, confirming its safety on chronic administration in mice. In HFHSD-fed mice supplemented with α5, we observed only a trend toward a reduction in plasma ALT levels, liver TG content, and some liver morphometric parameters (lipid area, fibrosis area, NAS). The failure to achieve significance could likely be attributed to the limited sample size or the high variability of the unsupplemented or α5-supplemented HFHSD groups for these parameters.

The unexpected reduction in liver CI activity observed in α5-treated mice under SPD conditions (but not under HFHSD) currently lacks a biological explanation. Importantly, the α5 supplement did not affect the activity of other MRC complexes in SPD-fed animals. Previous studies reported reduced CI and CIV activity in HFHSD-fed mice compared with chow-fed controls, which is partly consistent with our observations (Verbeek et al., 2015). As cellular respiration relies on coordinated MRC activity, selective changes in individual complexes should be viewed in this integrated context. Despite some conflicting results, evidence from both rodent and human studies suggests that mitochondrial alterations evolve during MASLD progression, with early adaptive adjustments in hepatic energy metabolism giving way to later-stage loss of metabolic flexibility (Fromenty and Roden, 2023). This dynamic complexity, compounded by variability in experimental models and conditions, makes the interpretation of liver MRC activity in diet-induced MASLD particularly challenging. In our study, no changes in markers of mitochondrial biogenesis (PGC-1α expression) or mass (citrate synthase activity, mtDNA content) were detected across the four experimental groups at the 17-week time point. Nevertheless, α5-supplementation was associated with significant increases in CII, CIII, CIV, and CV activity under HFHSD conditions, suggesting a diet-dependent effect. Further mechanistic studies will be required to elucidate the pathways underlying this α5-mediated modulation and its role in the progression of MASLD.

**FIGURE 7:**
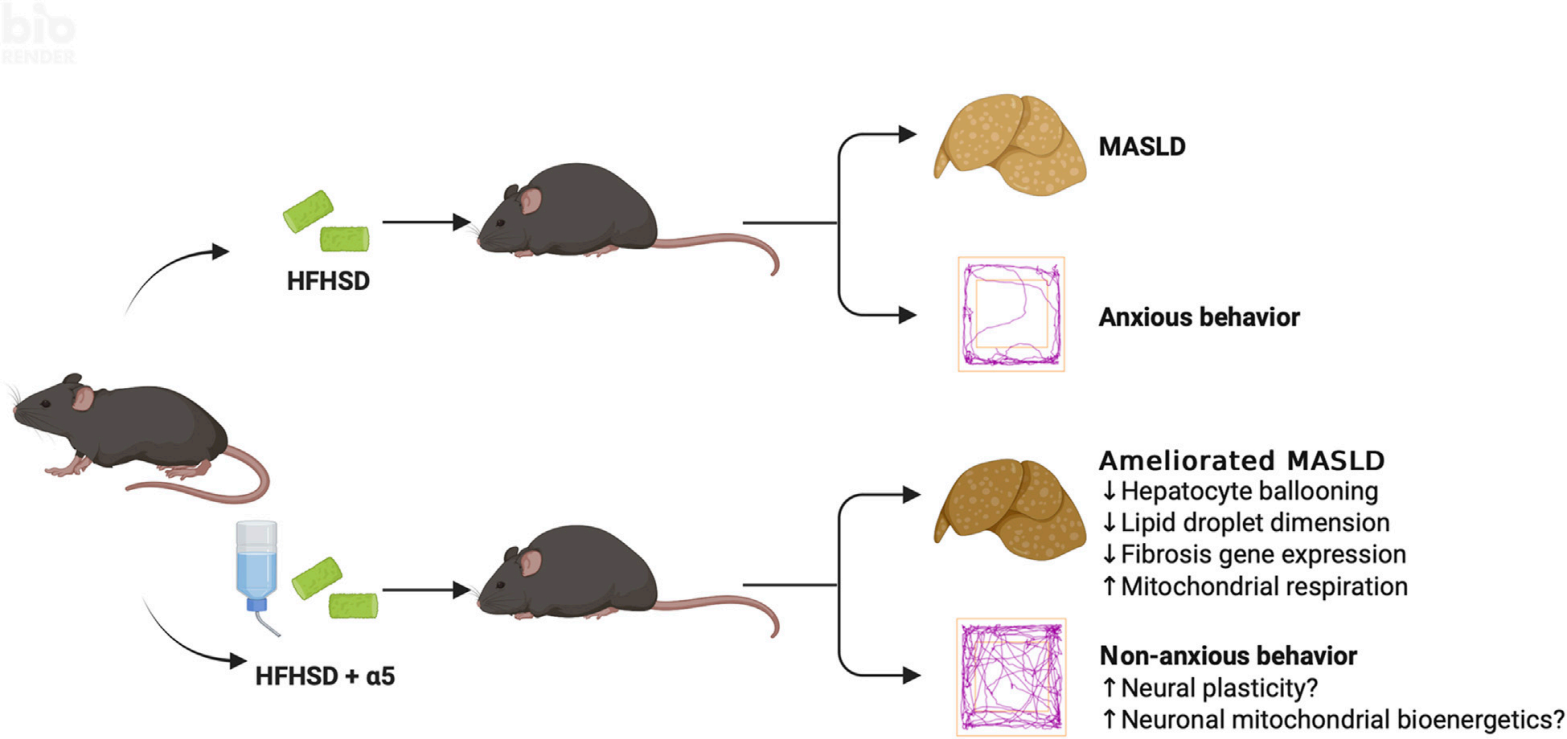
Conceptual schematic of the proposed effects of α5 supplementation. The diagram illustrates multiple mechanisms through which α5 may improve liver pathology in HFHSD-fed mice, independent of body weight. In parallel, α5 may modulate systemic and brain pathways, contributing to reduced anxiety-like behavior. Together, these actions support an integrated model in which α5 exerts protective effects across metabolic and neurobehavioral domains. Created with BioRender.com.

Except for increased Acox1 expression under SPD conditions, which may favor β-oxidation and catabolism of VLCFA, the compound did not affect the mRNA levels of genes involved in lipogenesis under HFHSD. Remarkably, dietary α5 supplementation significantly counteracted hepatocyte ballooning, an indicator of cellular damage and a prognostic factor for a greater risk of fibrosis progression (Singla et al., 2023). Further, α5 significantly reduced the number and diameter of lipid droplets in the liver from HFHSD-fed mice. Formerly considered inert markers of liver disease, lipid droplets indeed play active roles in MASLD pathophysiology, and their size correlates with the degree of fibrosis (Reid et al., 2024). Consistently, α5 treatment almost completely reversed the HFHSD-mediated increase in collagen transcripts, thereby strengthening the possibility of its favorable effect against hepatic fibrosis. Assays of fibrosis markers at the protein level may confirm these findings. Since the increased number and size of lipid droplets are associated with disease progression to steatohepatitis, strategies to modulate their biogenesis and growth are being proposed for their promising therapeutic potential (Bilson and Scorletti, 2024). Thus, α5 seems to act at one of the core mechanisms through which MASLD occurs and progresses (Figure 7). While further investigation should clarify α5-mediated mechanisms regulating lipid droplet size (including DGAT1, autophagy, or ER stress), it is worth noting that mitochondrial dysfunction is a key driver of disrupted lipid metabolism and fibrogenesis, which influence each other’s progression in MASLD.

Supplementation with BCAAs in liver disease has raised considerable interest, as well as controversy, due to the heterogeneity of the studies (Zhang et al., 2024). Previous work from our research group demonstrated that a specific EAA mixture enriched in BCAAs (BCAAem), unlike one based on the amino acid profile of casein, prevented liver steatosis, mitochondrial impairment, and oxidative stress in a rodent model of alcoholic liver disease (Tedesco et al., 2018). Liver protection was likely attributable to the BCAAem-mediated preservation of the mammalian/mechanistic inhibitor of rapamycin 1 (mTORC1) pathway, as found in ethanol-exposed HepG2 cells (Tedesco et al., 2018). Recently, other authors reported the multifaceted beneficial effects of metabolic modulators containing a specific combination of five amino acids in human cell model systems that mimic the NASH phenotype (Daou et al., 2021). The latter formula was well tolerated and decreased liver fat content in MASLD patients with or without type 2 diabetes (Harrison et al., 2021), encouraging further research in this field.

An additional relevant finding of the present study is the efficacy of dietary α5 supplementation in reducing MASLD-related anxiety, as revealed by the complete recovery of specific exploratory behaviors altered by HFHSD. We can only speculate about the potential mechanism(s) underlying the α5 psychotropic effect (Figure 7). We previously found that this compound promoted the full differentiation of murine and human neural stem cells into neuronal phenotypes, characterized by increased dendritic arborization and maturation of dendritic spines (Bifari et al., 2020). Enhanced mitochondrial energy metabolism, mediated by the activation of the mTORC1 and its downstream target p70 S6 kinase 1 (S6K1), appeared to be involved in these α5-mediated phenomena in newborn neurons (Bifari et al., 2020). Altered dendritic branching and spine density are observed in chronic stress and anxiety (McEwen et al., 2012), while neural plasticity is the target of conventional and emerging interventions to treat depression and anxiety disorders (Scangos et al., 2023). Interestingly, mice with genetic deletion of S6K1 displayed a robust anxiety-like behavior associated with reduced adult hippocampal neurogenesis (Koehl et al., 2021). The antioxidant capacity of α5 (Bifari et al., 2020; Dolci et al., 2022) could also contribute to its antianxiety effect. Finally, we cannot exclude the involvement of the gut-brain axis. Gut dysbiosis has been considered among the processes shared by MASLD and mental health disturbances (Shea et al., 2024). Therefore, modulation of gut microbiota, as observed with other EAA-based interventions (Ruocco et al., 2020) could have a role in both conditions.

This study has certain limitations. First, the small number of animals may have contributed to the lack of statistically significant changes in some parameters. Second, the α5-mediated mechanisms in MASLD improvement are not substantiated enough. Third, we cannot exclude the possibility that the observed mood alterations resulted from diet-induced obesity rather than being directly attributable to MASLD. Fourth, additional studies will be necessary to establish more precise correlations between HFHSD-induced liver disease and anxiety.

Our primary objective was to evaluate the efficacy of dietary α5 supplementation in ameliorating MASLD. While we also aimed to assess whether mice fed a Western diet would develop anxiety-like behaviors and whether EAA supplementation could mitigate these outcomes, the pronounced effect of the metabolic modulator on the anxiety phenotype was unexpected. This finding warrants further investigation to elucidate the underlying psychotropic mechanisms and to explore the potential efficacy of α5 supplementation in other models of mood disorders.

In conclusion, our data demonstrate that dietary supplementation with the EAA-based α5 designer formula, enriched with TCA cycle intermediates and cofactors, effectively counteracts the development of Western diet-induced MASLD in mice. Concurrently, α5 supplementation substantially reduced the anxiety behavior in this model. Notably, these beneficial effects occurred independently of changes in body weight or adiposity, suggesting that they are mediated by direct mechanisms activated by the specific combination of EAAs and metabolic enhancers. Further studies will be essential to fully characterize these mechanisms and to assess the translational potential of α5 supplementation in the management of metabolic and mood disorders.

## Data Availability

The raw data supporting the conclusion of this article will be made available by the authors, without undue reservation.
