# Supplementary material for: Dietary supplementation with a designer metabolic modulator improves MASLD and associated anxiety in mice
Source: Front Pharmacol. 2025 Oct 13;16:1661939. doi: 10.3389/fphar.2025.1661939 (PMC12554966; doi:10.3389/fphar.2025.1661939)
Supplement: Supplementary file 1 [file DataSheet1.pdf]

Dietary supplementation with a designer metabolic modulator improves MASLD and associated anxiety in mice

Supplementary Material

Table S1. Diet composition

	**Standard Purified Diet (SPD)**	**High Fat, High Sugar Diet (HFHSD)**
**Proteins**	12.4 g% - 13.7 kcal%	17.3 g% - 14.7 kcal%
**Carbohydrates**	68.3 g% - 75.9 kcal%	47.6 g% - 40.7 kcal%
**Fat**	4.1 g% - 10.3 kcal%	23.2 g% - 44.6 kcal%
**Kcal/g**	3,6	4,7
**Ingredients:**	**g/kg**	**g/kg**
**Casein**	140.0	195.0
**L-cystine**	1.8	3.0
**Corn starch**	465.7	56.9
**Maltodextrins**	155.0	60.0
**Anhydrous milk fat**	-	210
**Sucrose**	100.0	340.0
**Cellulose**	50.0	50.0
**Soybean oil**	40.0	20.0
**Mineral salts mix***	35.0	43.0
**Vitamine mix****	10.0	19.0
**Choline bitartrate**	2.5	3.0
**Antioxidant TBHQ**	0.008	0.04
**Food colorant**	-	0.1

* SPD: *AIN-93M-MX (94049); HFHSD***:** *AIN-93G-MX (94046)*
*** SPD & HFHSD: AIN-93-VX (94047)*
All diets were from Envigo, Italy

Table S2. Composition of the α5 formula

**Essential amino acids**	**g/100g**
L-Leucine	31.09
L-Lysine (chlorhydrate)	16.90
L-Isoleucine	10.36
L-Valine	10.36
L-Threonine	7.25
L-Cysteine	3.11
L-Histidine	3.11
L-Phenylalanine	2.07
L-Methionine	1.04
L-Tyrosine	0.62
L-Tryptophan	2.07
Vitamin B1 (thiamine chlorhydrate)	0.004
Vitamin B6 (pyridoxine chlorhydrate)	0.004
Citric acid	8.00
Malic acid	2.00
Succinic acid	2.00
Leucine: isoleucine: valine ratio	3:1:1

Table S3. Primers for qPCR

Gene	Primer sense	Primer antisense	Ta (°C)
Aacs	CCG TGT GGT CGG CTA TCT AC	TTT ACA CCA AAG TCC GGC GA	60
Acox1	CTT GCT GAA TCA GGG CAC CA	CAC AGA GCG TGG CCC ATC	60
Chop	CAC ACG CAC ATC CCA AAG CC	TCC TTC ATG CGT TGC TTC CC	63
Col1A1	GCC CTG CCG GAG AAG AAG GA	CCA CGG CTA CCA GGT CCA CC	63
Col6A1	GAT GAG GGT GAA GTG GGA GA	CAG CAC GAA GAG GAT GTC AA	63
Col6A2	CTT CCC CTA CCC CAA GTC TC	TGA TAT GGG GCT CTC AGG TC	60
Col6A3	ACG CCC ATC ACC ACT CTA AC	CTA AAC TGC ACG ACC CCA AT	60
Dgat2	CCG CAA AGG CTT TGT GAA G	GGA ATA AGT GGG AAC CAG ATC A	60
Eln	TCT TGC TCA ACC TCC TCC AT	CAA TAC CAG CCC CTG GAT AA	60
Fasn	TGC ACC TCA CAG GCA TCA AT	GTC CCA CTT GAT GTG AGG GG	60
Hprt	CTT CCT CCT CAG ACC GCT TTT	CAT CAT CGC TAA TCA CGA CGC	60/63
PGC-1α	AGC CGT GAC CAC TGA CAA CGA G	GCT GCA TGG TTC TGA GTG CTA AG	60
Co1	TGC TAG CCG CAG GCA TTA CT	CGG GAT CAA AGA AAG TTG TGT TT	60
Rnase P	GCC TAC ACT GGA GTC CGT GCT ACT	CTG ACC ACA CAC GAG CTG GTA GAA	60

Ta, temperature of annealing; *Aacs,* Acetoacetyl-CoA Synthetase; *Acox1,* Acyl-CoA Oxidase 1; *Chop,* C/EBP homologous protein; *Col1A1,* Collagen type I alpha 1 chain; *Col6A1,* Collagen type VI alpha 1 chain; *Col6A2,* Collagen type VI alpha 2 chain; *Col6A3,* Collagen type VI alpha 3 chain; *Dgat2*, Diacylglycerol O-acyltransferase 2; *Eln*, Elastin; *Fasn,* Fatty Acid Synthase; *Hprt,* hypoxanthine guanine phosphoribosyltransferase; *PGC-1α*, peroxisome proliferator-activated receptor γ coactivator 1α; *Co1*, Cytochrome c oxidase subunit I; *Rnase P*, Ribonuclease P.

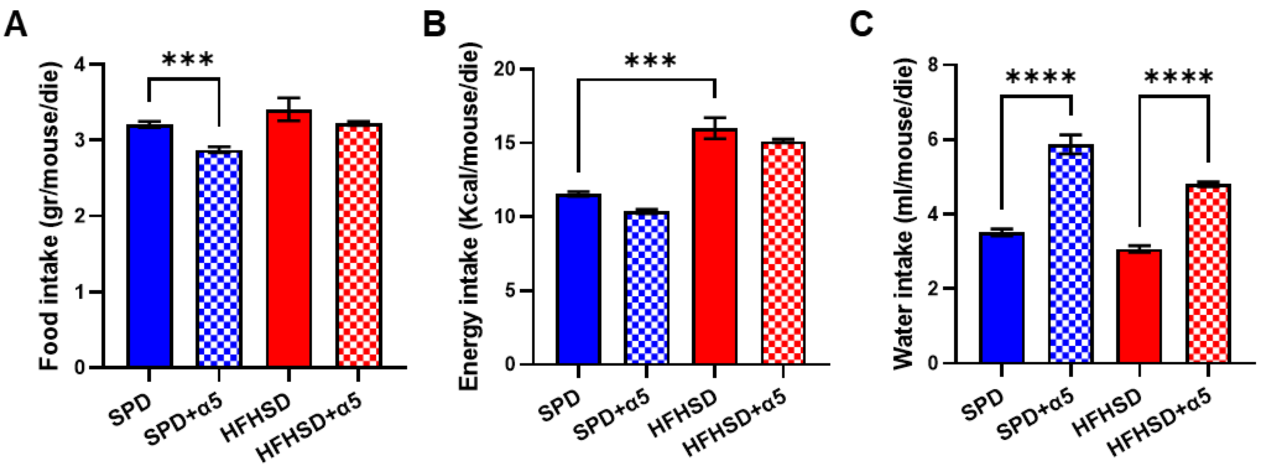

Figure S1. Effects of the 17-week nutritional interventions on average daily food (**A**), energy (**B**), and water (**C**) intake in C57BL/6J mice. Data represent mean ± SEM of weekly estimates (n=7-8 mice/group). SPD, standard purified diet; HFHSD, high-fat, high-sugar diet. Statistical analysis was performed by the Kruskal-Wallis test, followed by Dunn's multiple comparison test. *** p <0.001 and **** p<0.0001.

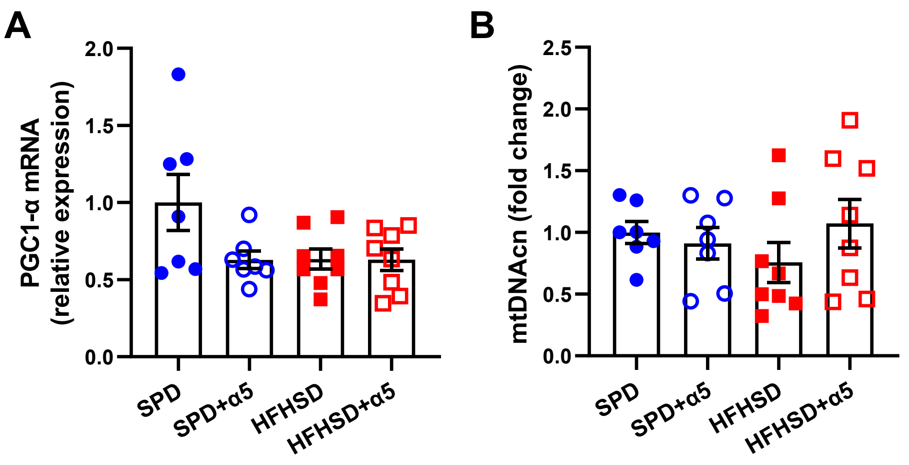

Figure S2. Relative *PGC1-α* mRNA expression in mouse liver (**A**). Reverse transcription-qPCR data were normalized to *Hprt* expression. Mitochondrial DNA copy number (mtDNAcn) (**B**). Data represent mean ± SEM (n=7-8 mice/group) and are reported as relative values compared to the SPD group, taken as 1. The statistical analysis was performed by one-way ANOVA, followed by Šídák's multiple comparisons test. *PGC1-α*, peroxisome proliferator-activated receptor γ coactivator 1α.

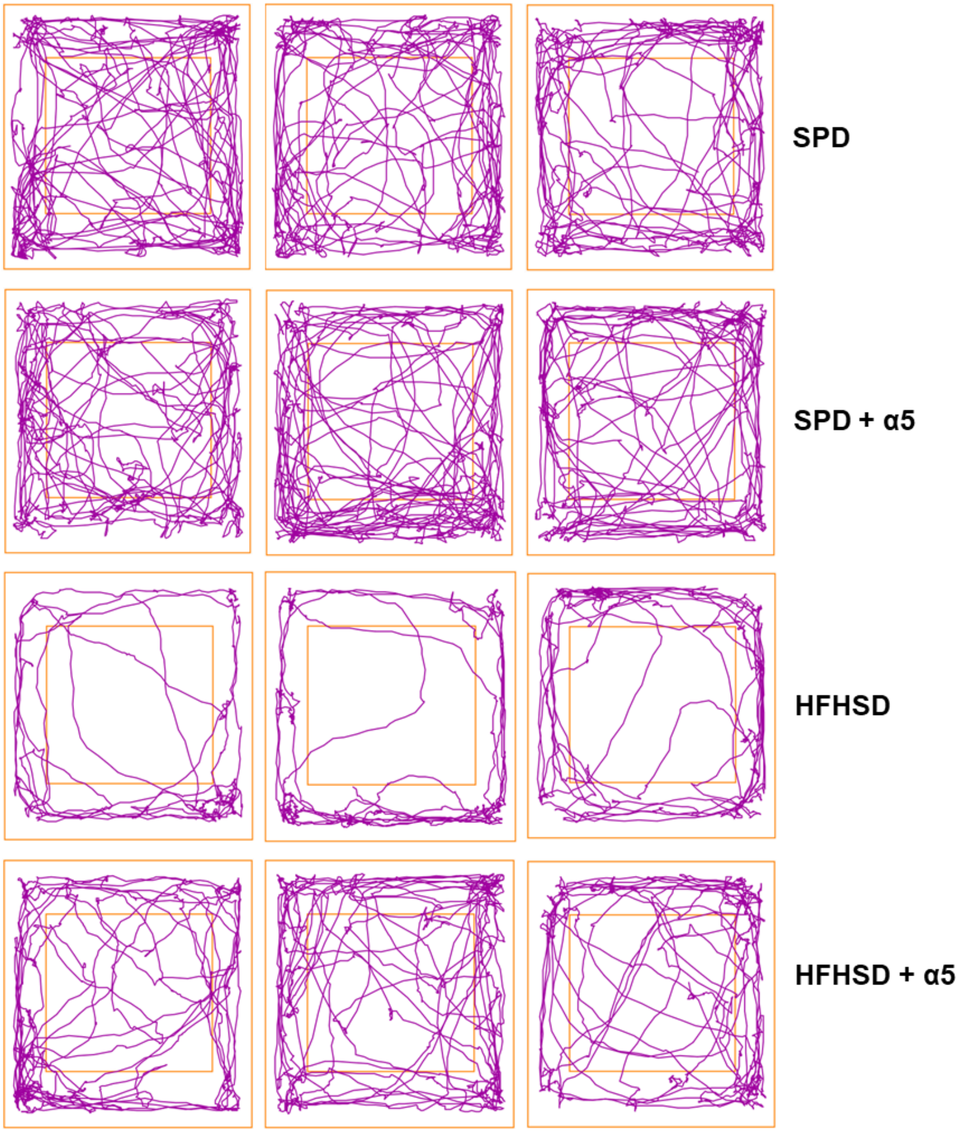

Figure S3. Open field test Path-tracking. Records of the total path travelled in the arena during the 5-minute test. Three representative images are shown per group.
